# Performance of the clinical assessment scale for autoimmune encephalitis in a pediatric autoimmune encephalitis cohort

**DOI:** 10.3389/fimmu.2022.915352

**Published:** 2022-10-14

**Authors:** Hao Zhou, Qun Deng, Zailan Yang, Zhaoqing Tai, Kaiyu Liu, Yue Ping, Yun Chen, Zhifeng Mao, Xiao Hu, Yi Wang

**Affiliations:** ^1^ Department of Pediatrics, Guizhou Provincial People’s Hospital, Medical College of Guizhou University, Guiyang, China; ^2^ Department of Pediatrics, Zunyi Medical University, Zunyi, China; ^3^ Department of Autoimmune Disease, Guangzhou KingMed Diagnostics Group Co., Ltd., Guangzhou, China; ^4^ Department of Neurology, Guizhou Provincial People’s Hospital, Guiyang, China; ^5^ Department of Neurology, Children’s Hospital of Fudan University, Shanghai, China

**Keywords:** autoimmune encephalitis, clinical assessment scale for AE, validity, reliability, children

## Abstract

**Background:**

The Clinical Assessment Scale for Autoimmune Encephalitis (CASE), a new scale used for rating the severity of autoimmune encephalitis (AE), has demonstrated good validity and reliability in adults with AE, but there is a shortage of data on its performance in children with AE. This study aimed to assess the reliability and validity of the CASE in a cohort of children with AE.

**Methods:**

Forty-seven pediatric inpatients with AE who visited Guizhou Provincial People’s Hospital between January 1, 2017, and October 31, 2021, were enrolled in the study. The CASE and mRS scores were obtained through a review of detailed medical records from the Health Information System by two pediatric neurologists. Finally, the performance of the CASE in this pediatric AE cohort was analyzed.

**Results:**

The results showed that anti-NMDA receptor encephalitis was the most common (61.70%) type of AE in children. The most common clinical manifestations were language problems (85.1%), psychiatric symptoms (80.9%), and dyskinesia/dystonia (78.7%). The CASE had good item reliability and interevaluator reliability; the Cronbach’s alpha value of the total score was 0.825, and the intraclass correlation (ICC) was 0.980. The Cronbach’s alpha value by item ranged from 0.16 to 0.406; items 1 and 9 had the lowest and highest Cronbach’s alpha values, respectively. The criterion validity between CASE and mRS total scores, as quantified by Pearson correlation, was 0.459, indicating slight to good criterion validity. The area under the curve (AUC) was 0.992 (95% confidence interval: 0.974-1.00). A cutoff value of 14 was selected to determine whether a patient needed admission to the ICU; this cutoff had a sensitivity of 100% and a specificity of 92%. The changes in EEG, MRI, and antibody titers were not related to the severity of AE. A CASE score cutoff of 9 was selected to indicate whether second-line treatment would be needed.

**Conclusion:**

The CASE has good reliability and validity in children with AE; however, some items of the CASE may not apply to this population. Thus, an in-depth study of the CASE is needed in children with AE.

## Introduction

Autoimmune encephalitis (AE) is a highly heterogeneous group of diseases whose main clinical characteristics are psychiatric symptoms, seizures, involuntary movements, and disturbance of consciousness (1). Patients with AE test positive for specific anti-neuronal antibodies, including antibodies against neuronal surface proteins, intracellular proteins, synaptic receptors, and ion channels. Since 2007, an increasing number of new specific anti-neuronal antibodies have been reported, and the study of AE has markedly expanded; in total, more than twenty extracellular antibodies and forty intracellular antibodies have been identified (2, 3). The estimated prevalence of AE in the United States and China is 0.8/100,000 and is increasing rapidly (4, 5). Previous studies have demonstrated that approximately 50% of AE patients require intensive care unit (ICU) treatment (6). Although most AE patients have a good prognosis and receive immunotherapy, some cases result in various lasting disabilities or even death (7). However, there is no single, specific biomarker to evaluate the prognosis of AE due to the highly heterogeneous clinical features of AE patients.

Clinical studies have shown that there are differences in the prognosis of AE patients with different clinical manifestations and autoimmune antibody types (8, 9). The contributing factors of poorer prognosis include older age, mental and behavioral disorders, movement disorders, disturbance of consciousness, central hypoventilation, and autonomic nervous dysfunction (10–12). These factors represent an increased severity of AE. The usual tool for severity of AE was the modified Rankin scale (mRS), the high score of mRS with the pool prognosis of AE (13). However, the mRS is a scale of global disability for acute stroke patients. The scale mainly focuses on motor function and assesses the ability to walk to evaluate functional independence (14). It is worth noting that AE patients present with a variety of clinical manifestations, including motor impairment, behavioral changes, seizures, memory deficits, and language disorders. Therefore, this scale is inappropriate for rating the severity of AE patients.

Recently, Professor Jung-Ah Lim developed a new scale used for rating the severity of autoimmune encephalitis (AE), named the Clinical Assessment Scale for Autoimmune Encephalitis (CASE) (15). One study demonstrated good validity and reliability in Chinese adults with AE (16), and another study included minor children with AE in a CASE validation study in Chinese AE patients (17). However, there are insufficient validation data for this new scale in children with AE. Previous studies have revealed that there are differences in clinical manifestations between children and adults with AE (18). Assessment of the validity of the CASE in children with AE is urgently needed. Therefore, this study aimed to assess the reliability and validity of the CASE in a cohort of children with AE.

## Methods

### Study populations

The clinical data of pediatric inpatients (under the age of 18) with AE who were hospitalized in Guizhou Provincial People’s Hospital from January 1, 2017, to October 31, 2021, were retrieved from the Health Information System (HIS) and retrospectively analyzed. All AE cases were diagnosed according to published diagnostic criteria (19), and antibodies were detected in both serum and CSF. The clinical data included age, sex, admission date, discharge date, age at onset, disease duration, clinical features, and treatment plans (as recommended by the guidelines for children with AE, first-line treatment for the present childhood AE cohort included IVGG and high-dose glucocorticoid or plasma exchange, and second-line treatment included rituximab and other immunosuppressants), as well as antibody test results, brain MRI findings, and EEG findings. Abnormalities on MRI were defined as T2 and FLAIR hyperintense lesions, etc.; abnormalities on EEG were defined as periodic or rhythmic patterns, diffuse or multifocal slow waves, generalized rhythmic delta activity, epileptic waves, etc.

## Assessment tool

### Clinical assessment scale for autoimmune encephalitis

The CASE was developed by Professor Jung-Ah Lim in 2019 (15). Dr. Cai et al. conducted a multicenter study to investigate the validation of the CASE in Chinese adults with AE (16). The CASE includes nine items, such as seizures (current status), memory dysfunction, psychiatric symptoms (delusion, hallucination, disinhibition, aggression), consciousness, language problems, dyskinesia/dystonia, gait instability and ataxia, brainstem dysfunction, and weakness. Each item was scored from 0 to 3 according to the severity of symptoms, with a total maximum score of 27 (15).

### The modified Rankin scale

To evaluate the criterion validity, we used the mRS as a criterion tool for children with AE. The modified Rankin scale (mRS) is a scale of global disability for acute stroke patients. The possible total scores range from a minimum of 0 to a maximum of 5. This scale is widely used for rating the severity of AE patients.

### Procedure

Two pediatric neurologists (Zailan Yang and Zhaoqing Tai, who have been working in pediatric neurology for almost ten years.) were responsible for the diagnosis evaluated the scales by reviewing the detailed medical records described from the HIS system, independently and blinded. The CASE and mRS were measured simultaneously on admission, admission to the PICU, and discharge. The study was conducted in accordance with the Declaration of Helsinki and approved by the institutional review board of Guizhou Provincial People’s Hospital. Patient consent was not available due to the retrospective nature of the study.

### Data analysis

Data analysis was performed by utilizing SPSS 26.0 (SPSS Inc., Chicago, IL). Cronbach’s alpha was employed to test the internal consistency of each item, with a value >0.70 considered to indicate good reliability. The interevaluator reliability was assessed by the interclass correlation coefficient (ICC). Pearson correlation analyses of CASE item scores and mRS total score to evaluate the criterion validity. Receiver operating characteristic (ROC) curves were generated, and 95% confidence intervals (CIs) were computed to determine the optimal cutoff value of the severity of AE. Severity was measured by admission to the ICU. The effects of the different clinical features on the CASE scores were compared by ANOVA. All tests were two-tailed, and a *P* value of less than 0.05 was considered statistically significant.

## Results

### Clinical data of the current pediatric AE cohort

Among the 47 children, 51.1% (24/47) were female, with an average age of 7.5 ± 3.5 years old; 61.70% (29/47) had NMDAR antibodies. Other types included Ma2, CASPR2, CV2, AMPAR1 and GABABR antibodies. Only 12.8% (6/24) had a history of infection in the early stage, but 82.9% had no obvious cause. The median length of hospital stay was 18 days (6-58 days); 53.2% (25/47) had abnormal MRI, and 87.2% (41/47) had abnormal EEG. All patients received immunoglobulin and high-dose hormonal therapy (20 mg/kg), and half (51.1%) required further care in the ICU. Antibodies were detected in the cerebrospinal fluid and serum of 53.2% (25/47) of children. The most common clinical manifestations were language problems (85.1%), psychiatric symptoms (80.9%), and dyskinesia/dystonia (78.7%), but gait instability and ataxia were rare (14.9%). The details are presented in **Table 1**.

**Table 1:** Demographic and clinical characteristics of enrolled patients with autoimmune encephalitis (N, mean ± SD, median, %).

Characteristics	Total	Antibody positive		Antibody negative
		NMDAR	Other types
**Number**	47	29	8	10
**Age (years)**	7.5 ± 3.5	7.6 ± 3.3	8.2 ± 3.5	6.7 ± 3.8
**Gender**				
Female	24 (51.1)	17 (58.6)	2 (25.0)	5 (50.0)
Male	23 (48.9)	12 (41.4)	6 (75.0)	5 (50.0)
**Inducement**				
Infection	6 (12.8)	1 (3.4)	1 (12.5)	4 (40.0)
Vaccine	2 (4.3)	0 (0.0)	0 (0.0)	2 (20.0)
Unknown	39 (82.9)	28 (96.6)	7 (87.5)	4 (40.0)
**Age at onset (years),** **median**	8 (1-13)	8 (2-12)	8 (2-13)	6 (1-13)
**Disease duration (days), median (min., max.)**				
From onset to Admission(days)	10 (1-120)	10 (1-60)	11 (2-120)	8 (4-32)
From admission to Diagnosis	2 (1-30)	2 (1-30)	1.5 (1-10)	1 (1-10)
From admission to immunotherapy	2 (1-14)	2 (1-14)	5 (2-11)	2 (1-4)
Hospital stays	18 (6-58)	17.5 (6-58)	23.5 (16-56)	18.5 (8-37)
**Clinical features, n (%)**				
Seizures	20 (42.6)	11 (37.9)	4 (50.0)	5 (50.0)
Memory dysfunction	35 (74.5)	22 (75.9)	6 (75.0)	7 (70.0)
Psychiatric symptoms	38 (80.9)	24 (82.8)	6 (75.0)	8 (80.0)
Conscious	29 (61.7)	21 (72.4)	3 (37.5)	5 (50.0)
Language problem	40 (85.1)	26 (89.7)	7 (87.5)	7 (70.0)
Dyskinesia/dystonia	37 (78.7)	25 (86.2)	6 (75.0)	6 (60.0)
Gait instability and ataxia	7 (14.9)	4 (13.8)	3 (37.5)	0 (0.0)
Brain stem dysfunction	14 (29.8)	9 (31.0)	3 (37.5)	2 (20.0)
Weakness	13 (27.7)	8 (27.6)	4 (50.0)	1 (10.0)
**MRI**				
Normal	22 (46.8)	12 (41.4)	5 (62.5)	5 (50.0)
Abnormal	25 (53.2)	17 (58.6)	3 (37.5)	5 (50.0)
**EEG**				
Normal	6 (12.8)	1 (3.4)	1 (12.5)	4 (40.0)
Abnormal	41 (87.2)	28(96.6)	7 (87.5)	6 (60.0)
**Immunotherapy**				
IVGG	47(100.0)	29 (100.0)	8 (100.0)	10 (100.0)
High dose glucocorticoid	47 (100.0)	29 (100.0)	8 (100.0)	10 (100.0)
plasma exchange	6 (12.7)	4 (13.8)	2 (25.0)	0 (0.0)
Rituximab	9 (19.1)	7 (24.1)	2 (25.0)	0 (0.0)
Others	2 (4.3)	2 (6.9)	0 (0.0)	0 (0.0)
**Admission to ICU, n (%)**				
Yes	24 (51.1)	15 (51.7)	5 (52.5)	4 (40.0)
No	23 (49.9)	14 (48.3)	3 (37.5)	6 (60.0)
**Presence of antibodies, n (%)**				
CSF	9 (19.1)	5 (17.2)	4 (50.0)	/
Serum	3 (4.3)	1 (3.4)	2 (25.0)	/
Both	25 (53.2)	23 (79.4)	2 (25.0)	/

### Reliability and validity of the CASE

The Cronbach’s alpha of the total score is 0.825, the Cronbach’s alpha of each item for the total score ranges from 0.16 to 0.406, and items 1 and 9 had the lowest and highest Cronbach’s alpha values, respectively. The interevaluator reliability was 0.994 (ICC: 0.993-0.976). In **Table 2**, the criterion validity with the mRS (Pearson correlations) was 0.459 between the two total scores. The correlation coefficient ranged from 0.312 to 0.9 between the score of each item and the mRS total score.

**Table 2:** Pearson correlation analyses of CASE scores and mRS total score.

CASE	mRS (total score)	
	r	*P*
**Item 1**	0.90	*0.549*
**Item 2**	0.723	*< 0.001*
**Item 3**	0.571	*< 0.001*
**Item 4**	0.541	*< 0.001*
**Item 5**	0.750	*< 0.001*
**Item 6**	0.619	*< 0.001*
**Item 7**	0.312	*0.033*
**Item 8**	0.386	*0.007*
**Item 9**	0.329	*0.024*
**Total score**	0.459	*0.002*

**Figure 1:**
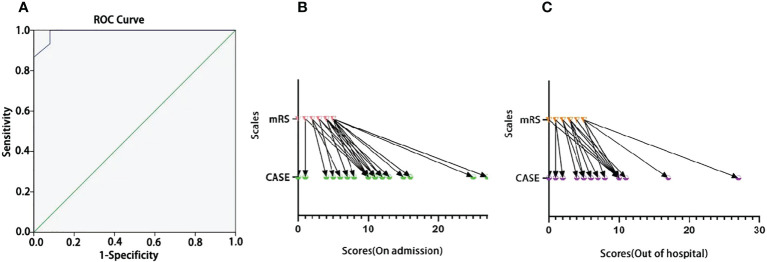
The ROC curve of the CASE total score as a predictor of disease severity in terms of the need for intensive care. **(A)** The one-to-one corresponding scores of the same individual on the CASE and the mRS [**(B)** for admission; **(C)** for out of hospital].

We tested the ability of the CASE to identify severity in terms of the need for intensive care. In **Figure 1A**, the AUC was 0.992 (95% CI: 0.974-1.00). The cutoff was 14, indicating that the patient needed admission to the ICU, with a sensitivity of 100% and specificity of 92%. **Figures 1B, C** show that the CASE is correlated with the mRS scale, and the scores of the same patient are consistent. However, CASE and mRS scores are given for the same patient on admission and out of the hospital (**Figures 1B, C** **)**, and the CASE has a wider range of possible scores than the mRS. This indicates that the mRS is less effective at capturing severity, such that it is difficult to see the severity and progression of the disease.

**Figure 2:**
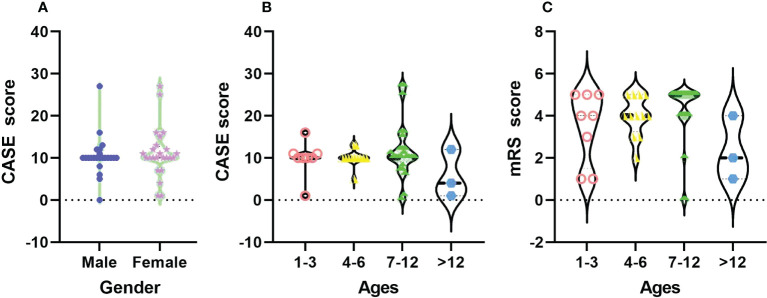
The distribution of CASE total scores among different sexes **(A)** and the total CASE **(B)** and mRS **(C)** scores by age at admission.

In **Figure 2**, there was no significant difference in CASE scores between male and female children with AE on admission (*F=0.244, P=0.624*). For different ages, the distribution of CASE scores was more extensive than that of mRS, and CASE was more able to comprehensively assess the real situation of patients. There was no significant difference in CASE scores on admission among the different age groups (*F=0.517, P=0.889*).

### Clinical features and CASE scores

**Figure 3:**
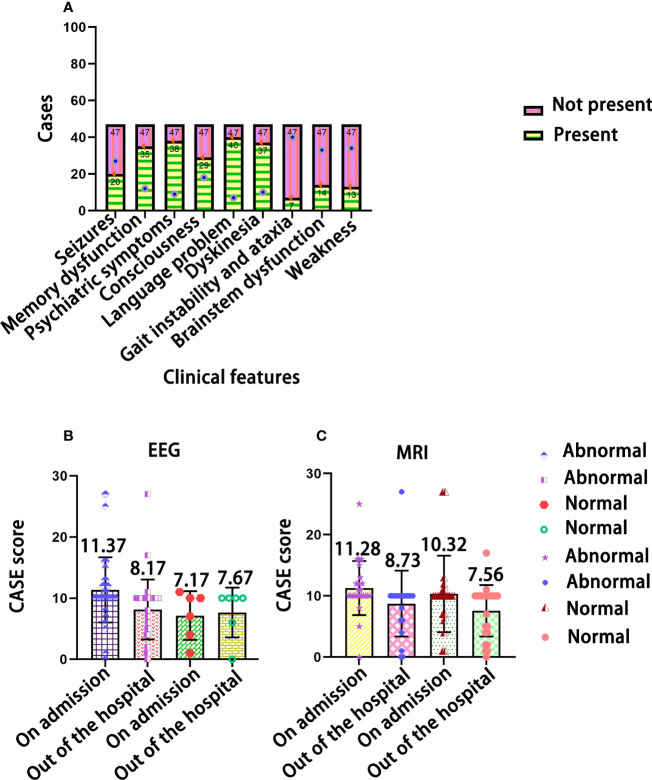
The distribution of clinical features in children with AE. **(A)** The relationship of CASE total scores with EEG **(B)** and MRI test results **(C)**.

In **Figure 3A**, the most common clinical manifestations were language problems (40/47) and psychiatric symptoms (38/47). In **Figures 3B, C**, after treatment, the CASE score of normal MRI and normal EEG at admission and discharge was not significant (*F=0.818, P=0.371; F = 0.222, P = 0.641*), but the changes in CASE score were more obvious in the children with abnormal MRI and abnormal EEG results after treatment (*F=9.317, P=0.004; F=8.000, P=0.006*).

### Treatment plans and CASE scores

**Figure 4:**
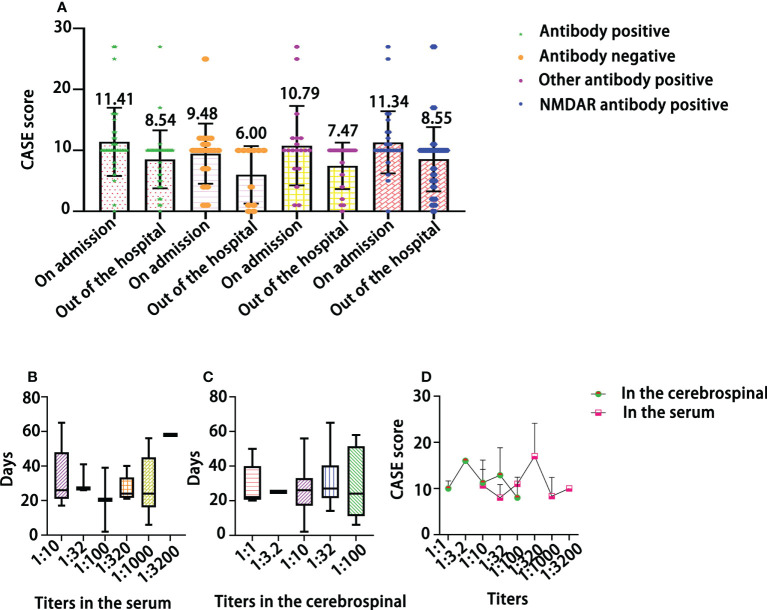
The relationship of CASE total scores with the results of antibody assays **(A)**, the length (days) of hospitalization by antibody titer in the serum **(B)** and in the cerebrospinal **(C)**, and the distribution of CASE scores by NMDAR antibody titer **(D)**.

As shown in **Figure 4A**, the CASE score did not significantly differentiate between patients who were antibody-positive on admission and those who were antibody-negative *(F=2.08, P>0.05*). The CASE score was decreased at discharge and was obviously lower in antibody-positive patients than in antibody-negative patients (*Ps<0.05*). There was no significant difference in length of stay among patients with different titers in their serum (**Figure 4B**) or their cerebrospinal fluid (**Figure 4C**) (*P>0.05*). Furthermore, there was no obvious association between NMDAR antibody titers and CASE scores (**Figure 4D**).

**Figure 5:**
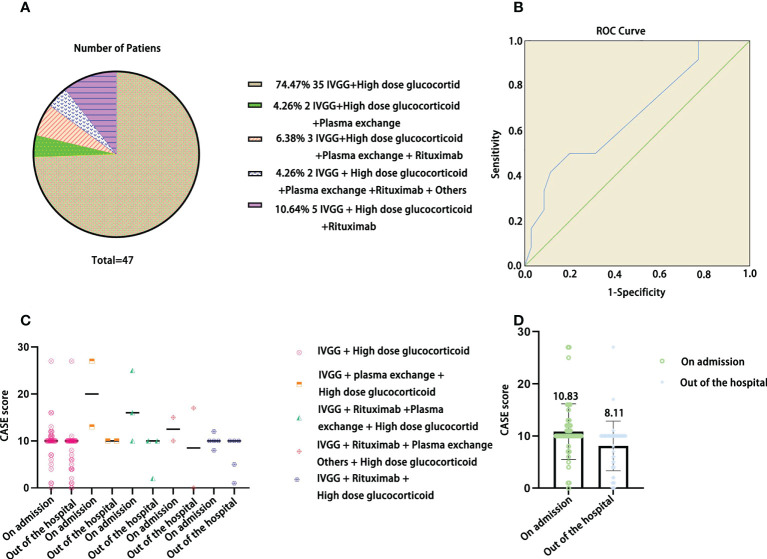
The distribution of treatment plans in the current pediatric AE cohort **(A)**, ROC curve of the CASE total score as a predictor of the patients who needed second-line treatment **(B)**, CASE scores at admission and discharge among different treatment plans **(C)**, and CASE total scores of the current pediatric AE cohort at admission and discharge **(D)**.

In **Figure 5A**, IVGG + high-dose glucocorticoid treatment was the most common (74.47%). In **Figure 5B**, we can see that the AUC was 0.673 (95% CI: 0.493-0.852), and a cutoff of 9 indicated that the patient needed second-line treatment, with a sensitivity of 91.7% and specificity of 77.1%. In **Figure 5C**, the CASE score was slightly decreased at discharge with different treatment plans. In **Figure 5D**, the CASE scores of children with AE were decreased after receiving different treatments at discharge compared with admission, but there was no significant difference *(F=6.832, P=0.10>0.05)*.

## Discussion

With the development of AE research, the number of children with AE has increased obviously (4). Anti-NMDAR encephalitis was the most common (61.70%) in our study, which is similar to previous studies (20). The most common clinical manifestations of AE in children are language problems and psychiatric symptoms, which were present in the current study and previous research (21). It is worth noting that the clinical symptoms in children with AE were different from those in adults with AE, and the most common clinical features were seizures, psychiatric symptoms, and memory dysfunction in adult AE patients (22). The CASE is a new scale for assessing the disease severity of AE. The development of the CASE is based on the clinical symptoms of adults with AE. Using this tool to assess the severity of children with AE, an analysis of reliability and validity is urgently needed.

The Cronbach’s alpha of the CASE total score is 0.825, and the results reveal that the CASE has good item reliability. However, the Cronbach’s alpha of some items for the total score was lower, especially item 1 (seizure). Comparatively, the Cronbach’s alpha of the CASE total score and each item was greater than 0.8 in adults in the AE cohort, the item of seizure with the highest Cronbach’s alpha value (15). This shows that the CASE was more suitable for adults with AE than children. Overall, the CASE scale is applicable to children, but not all items are applicable to them because the clinical manifestations of the disease are heterogeneous between children and adults (18). For example, seizure is the most common symptom in adults with AE, but it is relatively rare for children with AE. The interevaluator reliability was excellent. This indicates that most of these items are clearly evaluated and feasible. The CASE had slightly good criterion validity compared with the mRS between the two total scores. The value of the CASE in differentiating AE severity was excellent, and the AUC reached 0.992. A cutoff value of 14 had the highest sensitivity and specificity as an indicator that the patient needed admission to the ICU. The results show that the CASE is correlated with the mRS scale, and the scores of the same patient are consistent. However, the range of mRS scores was too small to highlight the severity of the patient, making it difficult to see the severity and progression of the disease. This scale is applicable to male and female students without significant difference. It should be noted that the consistency between the two scores is poor when the children are younger, which results in poor objectivity. Therefore, we can develop a slightly modified CASE for younger children, which is conducive to the evaluation of the real situation.

Many previous studies have demonstrated that clinical symptoms are related to the prognosis of AE (23). CASE total scores did not differ among patients with different MRI and EEG results on admission or outside the hospital, which indicates that the abnormal EEG and MRI results did not impact the AE severity. James Broadley et al. reported that MRI changes were unlikely to have significant prognostic value (24). Another study also revealed that seizure and EEG changes had no relationship with the prognosis of AE patients (25). The mean CASE score of the children with antibody positivity at admission was higher than that of the children with antibody negativity, indicating that the clinical manifestations of the children with antibody positivity were more obvious; the effect treatment was prominent in antibody-positive children. Some clinical research has shown that increased antibody titers may indicate relapse of AE (26); however, the antibody titers and prognosis of AE remain controversial. The results of this study illustrated that high antibody titers did not indicate more severe symptoms in AE patients, and the relationship between antibody titers and CASE scores needs more research in the future. All pediatric AE patients received IVGG + high-dose glucocorticoid treatment. A CASE score above a cutoff value of 9 indicated an increased probability that a patient would need second-line treatment.

### Limitations

Our study is the first to describe the reliability and validity of the CASE scale for children. The evaluation included only a small dataset, leaving some of the results prone to bias, and some conclusions need to be verified by more data. In particular, data for the youngest age group are scarce, such that the disadvantages of the CASE scoring scale for infants and young children are not well understood, but the evaluators obviously feel the difficulties in using the CASE to assess the severity in younger children with AE.

## Conclusions

In general, the CASE has good reliability and validity in children with AE. The CASE can assess the severity of patients’ disease and prognosis more effectively than the mRS can. However, some items of the CASE may not apply to children with AE. Thus, an in-depth study of the CASE is needed in children with AE.

## Data availability statement

The original contributions presented in the study are included in the article/supplementary material. Further inquiries can be directed to the corresponding authors.

## Ethics statement

The studies involving human participants were reviewed and approved by Guizhou Provincial People’s Hospital. Written informed consent from the participants’ legal guardian/next of kin was not required to participate in this study in accordance with the national legislation and the institutional requirements.

## Author contributions

HZ and YW conceived of the study. HZ, QD and ZY contributed to the analysis, synthesis and interpretation of the results and wrote the manuscript. ZT, KL, YP, YC, ZM, and XH contributed to the data collection. All authors contributed to the preparation of the manuscript. All authors contributed to the article and approved the submitted version.

## Funding

This project was supported by the National Natural Science Foundation of China (NSFC, 81860280).

## Acknowledgments

We sincerely thank all the patients who participated in this study, as well as the clinicians who contributed to the diagnosis and treatment of the children with AE.

## Conflict of interest

Author ZM was employed by Guangzhou KingMed Diagnostics Group Co., Ltd.

The remaining authors declare that the research was conducted in the absence of any commercial or financial relationships that could be construed as a potential conflict of interest.

## Publisher’s note

All claims expressed in this article are solely those of the authors and do not necessarily represent those of their affiliated organizations, or those of the publisher, the editors and the reviewers. Any product that may be evaluated in this article, or claim that may be made by its manufacturer, is not guaranteed or endorsed by the publisher.
